# MPP^+^ decreases store-operated calcium entry and TRPC1 expression in Mesenchymal Stem Cell derived dopaminergic neurons

**DOI:** 10.1038/s41598-018-29528-x

**Published:** 2018-08-06

**Authors:** Yuyang Sun, Senthil Selvaraj, Sumali Pandey, Kristen M. Humphrey, James D. Foster, Min Wu, John A. Watt, Brij B. Singh, Joyce E. Ohm

**Affiliations:** 1 Department of Biomedical Sciences, School of Medicine and Health Sciences, University of North Dakota, Grand Forks, North Dakota 58203 USA; 2 Biosciences Department, Minnesota State University, Moorhead, Moorhead, MN USA; 3 Department of Cancer Genetics and Genomics, Roswell Park Cancer Institute, Buffalo, NY 14263 USA; 4 School of Dentistry, UT Health Science Center San Antonio, TX 78229 San Antonio, USA

## Abstract

Parkinson’s disease is a neurodegenerative disorder involving the progressive loss of dopaminergic neurons (DNs), with currently available therapeutics, such as L-Dopa, only able to relieve some symptoms. Stem cell replacement is an attractive therapeutic option for PD patients, and DNs derived by differentiating patient specific stem cells under defined *in-vitro* conditions may present a viable opportunity to replace dying neurons. We adopted a previously published approach to differentiate Mesenchymal Stem Cells (MSCs) into DN using a 12-day protocol involving FGF-2, bFGF, SHH ligand and BDNF. While MSC-derived DNs have been characterized for neuronal markers and electrophysiological properties, we investigated store-operated calcium entry (SOCE) mechanisms of these DNs under normal conditions, and upon exposure to environmental neurotoxin, 1-methyl, 4-phenyl pyridinium ion (MPP^+^). Overall, we show that MSC-derived DNs are functional with regard to SOCE mechanisms, and MPP^+^ exposure dysregulates calcium signaling, making them vulnerable to neurodegeneration. Since *in-vitro* differentiation of MSCs into DNs is an important vehicle for PD disease modeling and regenerative medicine, the results of this study may help with understanding of the pathological mechanisms underlying PD.

## Introduction

Parkinson’s disease (PD) involves a progressive loss of DNs, and is the second most common chronic neurodegenerative disease after Alzheimer’s disease. PD prevalence is age associated, increasing significantly in incidence above the age of 65^1,2^. The prevalence rates are likely to increase as the population ages^3^, and by 2040, the annual cost to US society is expected to exceed $50 billion^4^. PD drastically affects quality of life as patients develop motor symptoms, such as bradykinesia, resting tremor, shuffling gait, rigidity, flexed posture, freezing, loss of postural reflexes, and non-motor symptoms, such as depression, lack of motivation, passivity and dementia.

The cause of majority of PD cases remains unknown. Although numerous genes and genetic loci have been implicated^5^, only a few percent of PD cases involve mutations in single genes, and 90% of PD patients have no family history of PD^6^. Epidemiologic studies have implicated numerous environmental risk factors in PD pathogenesis^7,8^. One such environmental risk factor was identified by Langston and colleagues as 1-methyl-4-phenyl-1, 2, 3, 6-tetrahydropyridine (MPTP)^9^. MPTP is formed by the dehydration of an intermediate compound required for the synthesis of an active narcotic compound, 1-methyl-4-propionoxypyridine (MPPP). Admixed with MPPP, MPTP was sold as “synthetic heroin”, which resulted in PD symptoms and ER visits by intravenous drug users of San Jose, California in 1982^10^. Now, MPTP is commonly used to recapitulate some element of PD pathology in cell-culture based and animal models of PD.

Pathologically, the hallmarks of PD are the death of DNs in the substantia nigra pars compacta, and the presence of cytoplasmic protein aggregates, known as Lewy bodies. The cellular dysfunctions underlying these pathological hallmarks include abnormal mitochondrial metabolism, oxidative stress, neuroinflammation and upregulation of unfolded protein response (UPR) in the endoplasmic reticulum (ER)^11,12^. One of the most important signaling molecules, and a common denominator in these cellular dysfunctions, is intracellular calcium [Ca^2+^]_i_^13,14^. Ca^2+^ levels are tightly controlled throughout neurons, including within major organelles such as mitochondria and ER. Ca^2+^ regulates several cellular functions such as enzyme phosphorylation and dephsophorylation, cytoskeletal mediated motility, neurotransmitter release, transcription of numerous genes, and the process of programmed cell death. Ca^2+^ signaling depends on increased levels of intracellular Ca^2+^ derived either from outside the cell or from stores within the ER. Alterations in Ca^2+^ levels may occur due to excessive Ca^2+^ influx in the cytosol, overloading of mitochondria with Ca^2+^or depletion of ER Ca^2+^ stores.

ER plays an important role in Ca^2+^ homeostasis. Several proteins regulate Ca^2+^ movement across the ER membrane, and includes IP_3_ receptors which serve to release Ca^2+^ from the ER in response to cell surface stimulation^15,16^. A plant toxin, called Thapsigargin, also releases the same pools of Ca^2+^, as does IP_3_^17^. Upon ER Ca^2+^ store depletion, store-operated Ca^2+^ entry (SOCE) mechanisms in the plasma membrane are believed to be activated to replenish intracellular stores in ER. Influx through this pathway may provide direct Ca^2+^ signals to localized, spatially restricted target areas close to the sites of Ca^2+^ entry, thus initiating specific signaling pathways^18^ important for neurosecretion, sensation, long-term potentiation or depression of synaptic transmission, synaptic plasticity, gene regulation, neuronal growth and differentiation^19^. While the molecular identity of SOCE has not been conclusively identified, especially in neuronal cells, the transient receptor potential canonical (TRPC) channels, even if not direct components, interact with them^19–21^. These channels are important in maintaining ER Ca^2+^ levels, and movement of the ER away from its Ca^2+^ set point can compromise proteostasis, thereby triggering UPR, and ultimately neuronal death^21–24^.

The current treatment strategies for PD rely mostly on pharmacologic replacement of dopamine (DA) with L-dopa, which provides some symptomatic relief, but does little to reverse or slow down the progression of the disease^25,26^. In this regard, cell-based treatments to restore nigrostriatal neuronal function by transplanting cells with DA-producing capacity into the striatum holds great potential for PD. Several cellular sources have been investigated for the potential to act as renewable supply of transplantable dopaminergic neurons for PD, including fetal brain tissue, embryonic stem cells, induced pluripotent stem cells, neural stem cell precursors, and mesenchymal stem cells (MSCs)^27^.

MSCs are multi-potent stem cells, with the ability to proliferate, symmetrically divide and produce multi-lineage mesodermal and non-mesodermal derivatives *in vitro* and *in vivo*^27^ including DNs^28–41^. These reports of differentiation, along with those of MSC-mediated disease improvement in experimental animal models of PD^41,42^, have garnered much interest in the use of MSC-derived DNs as therapeutics to replace dying neurons in specific brain regions of PD patients. Several differentiation protocols have thus far been adapted, which have characterized the MSC-derived DNs to varying extents; however, a complete characterization of these neurons is still missing. Before the proposed stem-cell based therapy can effectively translate into the clinic, it is important to fully understand the function of MSC-derived DNs under physiological and pathological conditions. Towards this end, we investigated Ca^2+^ signaling, specifically SOCE mechanisms, TRPC1 expression, and the effect of MPP^+^, an environmental neurotoxin and a proposed risk factor for PD on SOCE and TRPC1 expression in these neurons.

## Material and Methods

### Neuronal differentiation of Mesenchymal Stem Cells (MSCs)

**Figure 1:**
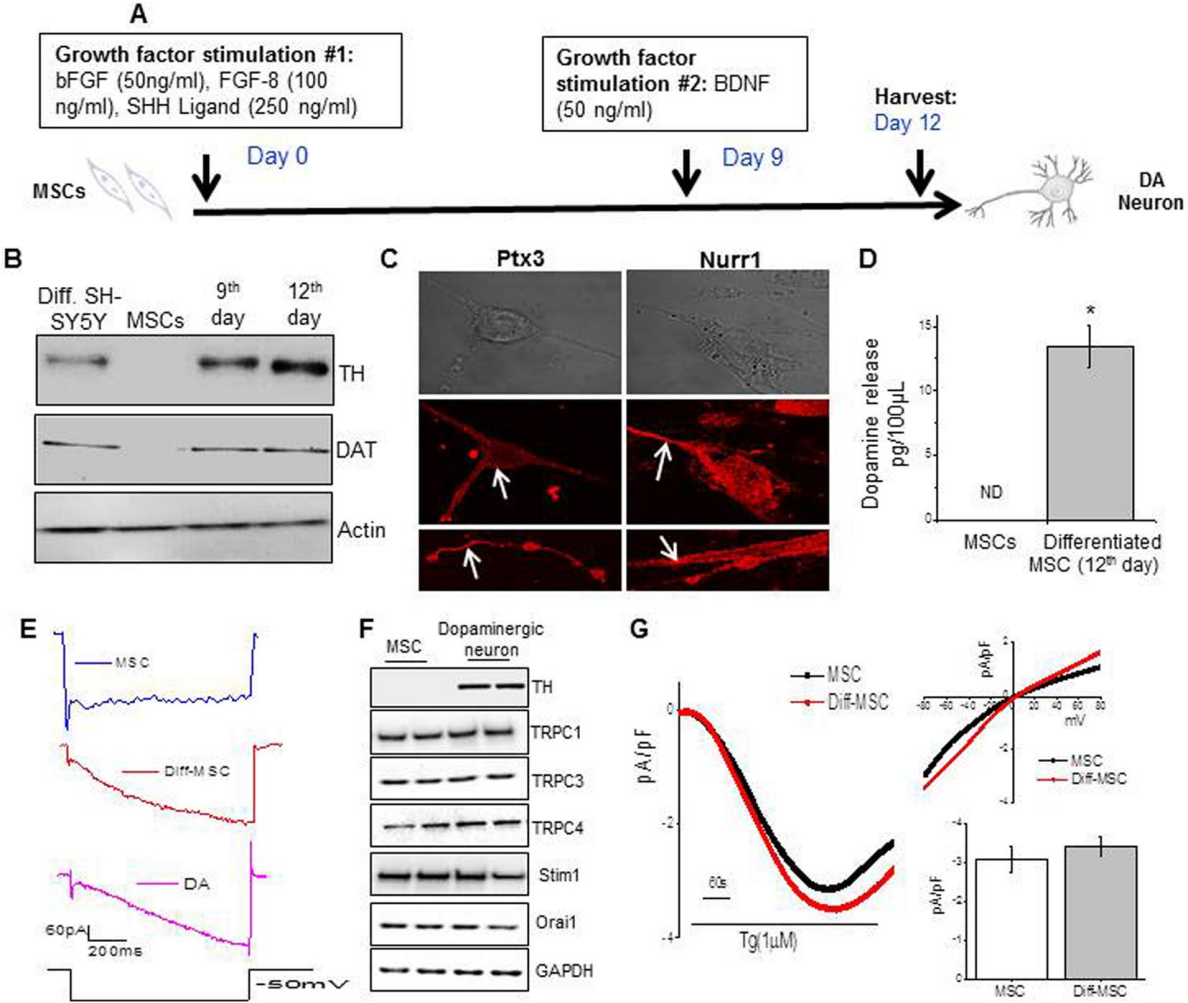
MSCs induced with growth factors show characteristics of dopaminergic neurons. MSCs were induced with growth factors; b-FGF, FGF-8 and SHH ligand for 9 days, followed by addition of BDNF for next 3 days (**A**). The induced MSCs were analyzed for dopaminergic neuron characteristics on day 12 of differentiation. TH and DAT protein expression was analyzed in the protein lysate obtained from SH-SY5Y (induced into neuronal like cells with retinoic acid), un-induced MSCs and induced MSCs on day 9 and 12 of differentiation, via western blot (**B**). Ptx3 and Nurr1 expression was analyzed in differentiated MSCs via immunofluorescence (**C**). Dopamine release was quantified in the supernatant of MSCs and differentiated MSCs on day 12 of differentiation via ELISA (**D**). Bars represent mean ± SE. *p value < 0.05, as compared with MSCs (**D**). The electrical signature of MSCs and differentiated MSCs (**E**) and in mouse dopaminergic slices indicated that a hyperpolarizing pulse of 60 mV induced a large inward current in differentiated MSCs. Western blots showing expression of proteins involved in calcium entry are shown in (**F**). Antibodies used for detection are labeled in the figure. Traces of Tg (1 µM) induced currents were measured in MSCs and differentiated MSCs (**G**). The holding potential for current recordings was −80 mV and the relative current-voltage (I–V) curves along with the current density (average from 7–10 cells) is shown as bar graph.

Bone-marrow derived MSCs were obtained from Lonza Group Ltd. (Basel, Switzerland), and maintained in culture, as per manufacturer’s instructions. For dopaminergic neuronal differentiation of MSCs, Trzaska K. *et al*.’s protocol was adopted^43^, and is depicted in Fig. 1A. Briefly, MSCs were plated in MSC-culture media, and allowed to adhere to Poly-D-lysine coated cell culture vessel’s surface at 37 °C for 24 hours, in a 5% CO_2_ incubator. Neuronal differentiation was initiated by replacing the MSC-culture media with neurobasal media supplemented with 0.5% B27 (Invitrogen^TM^, Life Technologies, Carlsbad, CA, USA), 250 ng/ml SHH, 100 ng/ml FGF-8, and 50 ng/ml bFGF (R&D Systems, Inc., Minneapolis, MN). The cells were maintained in the same media throughout the 12-day differentiation protocol. On day 9 of differentiation, 50 ng/ml BDNF was spiked into the differentiation media. Undifferentiated MSCs were maintained as negative controls, and SH-SY5Y cells (American Type Culture Collection, Manassas, VA, USA) differentiated into neurons with the help of retinoic acid (10 µM for 7 days), were used as positive controls in the functional characterization experiments.

### Drug treatment

At the end of 12-day differentiation protocol, cells were treated with 500 µM MPP^+^ (Sigma-Aldrich Co. LLC, St. Louis, MO, USA) or corresponding dilution of dimethylsulfoxide (vehicle control) for times indicated in the results or figures. For calcium measurements, IP_3_ (10 µM) or Thapsigargin (1 or 2 µM) was added just before gathering the data. For non-specifically blocking the Transient Receptor Potential Cation (TRPC) channel, cells were pre-treated with 100 µM SKF96365 for 30 mins.

### Western Blot analyses

At the end of 12-day differentiation protocol, cells were harvested and stored at −80 °C, until use. Whole cell lysates were prepared in RIPA lysis buffer supplemented with protease and phosphatase inhibitors. Total protein concentrations were determined using Bradford reagent (Bio-Rad Laboratories, Inc., Hercules, CA, USA), resolved on NuPAGE 4–12% Bis-Tris gels (Invitrogen^TM^, Life Technologies, Carlsbad, CA, USA), transferred to polyvinylidene difluoride (PVDF) membranes, and probed with respective antibodies. A 1:500 dilution of the primary antibody (Cell Signaling Technology, Inc., Beverly, MA) was used to probe for Tyrosine Hydroxylase (TH) and Dopamine active transporter (DAT). The remaining antibodies were used at a 1:1000 dilution. Corresponding peroxidase-conjugated secondary antibodies (Sigma-Aldrich Co. LLC, St. Louis, MO, USA), ECL reagent (Thermo Fisher Scientific, Waltham, MA, USA) and Quantity One Analysis software (Bio-Rad Laboratories, Inc., Hercules, CA, USA) were used to detect the protein bands.

### Dopamine (DA) quantification

DA levels were quantified using a commercially available Enzyme-Linked Immunosorbent Assay (ELISA, Rocky Mountain Diagnostics, Colorado Springs, CO, USA). For dopamine release, cells (differentiated or undifferentiated MSCs or those treated with SKF96365) were depolarized with 50 mM of K^+^ solution. 100 µl supernatant was collected and DA level was measured immediately, as per manufacturer’s instructions.

### Calcium measurements

The intracellular calcium concentrations were measured as previously described^24^. Differentiated MSCs, after treatment with MPP^+^ for 2, 4, 6 12, and 24 hours, were incubated with 2 µM Fura-2 (Molecular Probes^®^, Life Technologies, Carlsbad, CA, USA), a calcium indicator, for 45 mins. at 37 °C, in a 5% CO_2_ incubator. After washing the cells with Ca^2+^ free buffer (10 mM HEPES, 120 mM NaCl, 5.4 mM KCl, 1 mM MgCl_2_, 10 mM glucose, pH 7.4), the fluorescence intensity of Fura-2 loaded control cells was monitored with a CCD camera-based imaging system (Compix Inc., Imaging Systems, Township Cranberry, PA, USA) mounted on an Olympus XL70 inverted microscope, equipped with an Olympus 40x (1.3 NA) objective lens. A monochrometer dual wavelength enabled alternative excitations at 340 and 380 nm, whereas the emission fluorescence was monitored at 510 nm with an Okra Imaging camera (Hamamatsu, Japan). The images of multiple cells, collected at each excitation wavelength, were processed using the C imaging, PCI software (Compix, Inc., Imaging Systems, Township Cranberry, PA, USA) to provide *F340/F380 nm* fluorescence ratios. The analog plots represent the intracellular ([Ca^2+^]_i_) values averaged from 30–40 cells, and are representative results from 3–4 separate experiments.

### Electrophysiology

Cell-attached patch clamp measurements were performed, as previously described^44^. Coverslips with cells were transferred to the recording chamber and perfused with external Ringer’s solution (145 mM NaCl, 5 mM KCl, 1 mM MgCl_2_, 1 mM CaCl_2_, 10 mM HEPES, 10 mM glucose, pH 7.4 with NaOH base). Whole cell currents were recorded in a tight-seal whole cell configuration at RT, using Axopatch 200B amplifier (Molecular Devices, LLC, Sunnyvale, CA, USA). The patch pipette had resistances between 3 and 5 MΏ after filling with the standard intracellular solution (150 mM cesium methane sulfonate, 8 mM NaCl, 10 mM HEPES, 10 mM EGTA, ph 7.2 with CsOH base). Voltage ramps ranging from −100 mV to +100 mV, at a holding potential of 0 mV were delivered to the cells, for 100 ms, at 2 s intervals. Currents were recorded at 2 kHz and digitized at 5–8 kHz. pClamp 10.1 software (Molecular Devices, LLC, Sunnyvale, CA, USA) was used for data acquisition and analysis. Basal leak was subtracted from the final current measurements, and average currents are shown.

### Immunofluorescence

Cells were grown on coverslips, washed twice with phosphate-buffered saline (PBS), and fixed for 30 min using 3% paraformaldehyde. The cells were permeabilized using cold methanol and blocked for 20 mins using 5% donkey serum. Cells were probed with the primary antibodies (anti-TRPC1, anti-TH, anti-Nurr1 and anti-Ptx3) at a 1:100 dilution for 1 h. After washing thrice with PBS + 0.5% bovine serum albumin, the cells were labeled with rhodamine or FITC conjugated anti-rabbit secondary antibodies (The Jackson Laboratory, Bar Harbor, Maine, USA) at a 1:100 dilution. Confocal images were collected using a MRC 1024 krypton/argon laser scanning confocal equipped with a Zeiss LSM 510 Meta photomicroscope (Carl Zeiss, Oberkochen, Germany).

### Cell viability

Cells were seeded in 96-well plates at a density of 0.5 × 10^6^ cells/well. The cultures were pretreated with 100 µM SKF96365 for 30 mins. Cell viability was determined using Vybrant^®^ MTT cell proliferation assay kit (Life Technologies, Carlsbad, CA, USA), as previously described^44^. 30 µl of MTT reagent (0.5 mg/ml MTT in PBS containing 10 µM HEPES) was added to each well, and cells were incubated in a CO_2_ incubator for 2 h. The medium was aspirated from each well, and the culture plate was dried at 37 °C for 1 h. The resulting formazan dye was extracted with 100 µl of 0.04 N HCl in isopropanol, and the absorbance was measured in a micro plate reader (Molecular Devices, LLC, Sunnyvale, CA, USA) at 570 and 630 nm. Cell viability was expressed as a percentage of the control (untreated MSC-derived dopaminergic neurons).

### Statistical analysis

All results are expressed as mean ± S.E. Origin 7.0 (Origin Lab Corporation, Northampton, MA, USA) was used to calculate statistics; differences between groups were tested with two-tailed, paired student’s t-test. In all cases, P ˂ 0.05 was considered statistically significant.

### Availability of Data and Materials

Materials, data and associated protocols will be promptly made available to the editorial board and readers without undue qualifications in material transfer agreements as outlined in the Scientific Reports Editing and Publishing Policies.

## Results

### Characterization of dopaminergic neurons derived from Mesenchymal Stem Cells (MSCs)

We attempted differentiation of MSCs into dopaminergic neurons (DNs) using several published protocols, and found that the one reported in this paper (Fig. 1A), worked the best in our hands. MSC-derived DNs were characterized based on their morphology, protein expression and function. The neural morphology was indicated due to the refractive appearance of the cells, and presence of bipolar and multipolar neurite like projections. Uninduced MSCs did not show expression of neural-specific proteins; Tyrosine hydroxylase (TH) or Dopamine Active Transporter (DAT) (Fig. 1B). However, like SH-SY5Y cells induced with retinoic acid, the MSCs induced with growth factors showed increased expression of TH and DAT by day 9 of differentiation, and a prominent expression by day 12 of differentiation (Fig. 1B). The expression of Ptx3 and Nurr1, which are the markers for dopaminergic neurons, were also significantly increased in the MSC-derived DNs, and their expression was observed in the soma as well as in the dendrites (Fig. 1C). Unlike un-induced MSCs, MSC-derived dopaminergic neurons were functional, and this was assessed by their capability to release dopamine upon stimulation with KCl (Fig. 1D) and generate *I*_h_ currents which was induced by a hyperpolarizing pulse of 60 mV for 1.5 s duration (in voltage clamp) (Fig. 1E) that were similar to that observed in dopaminergic neurons. Taken together, these results show that using the differentiation approach described here (Fig. 1A), we could obtain DNs, which are phenotypically and functionally similar to DNs.

### MPP^+^ decreases store mediated Ca^2+^ entry in MSC-derived dopaminergic neurons

The neuronal Ca^2+^ concentration is tightly regulated, and disturbances in Ca^2+^ homeostasis have been implicated in several neuropathological conditions^20^. Alterations in Ca^2+^ homeostasis may be caused by exposure to MPP^+^, which in turn contributes to loss of dopaminergic neurons via apoptosis^21^. We initially investigated the expression of various calcium entry channels in both control MSCs and 12 day post differentiation of MSCs. As shown in Fig. 1G TRPC1, TRPC3, TRPC4, Orai1, and STIM1 were all expressed in un-induced MSCs and no further change in their expression was observed when compared with differentiated cells (Fig. 1F). To evaluate the calcium entry channel we performed electrophysiological recordings which showed a liner current and was also similar in both un-induced MSCs and differentiated MSCs (Fig. 1G).

**Figure 2:**
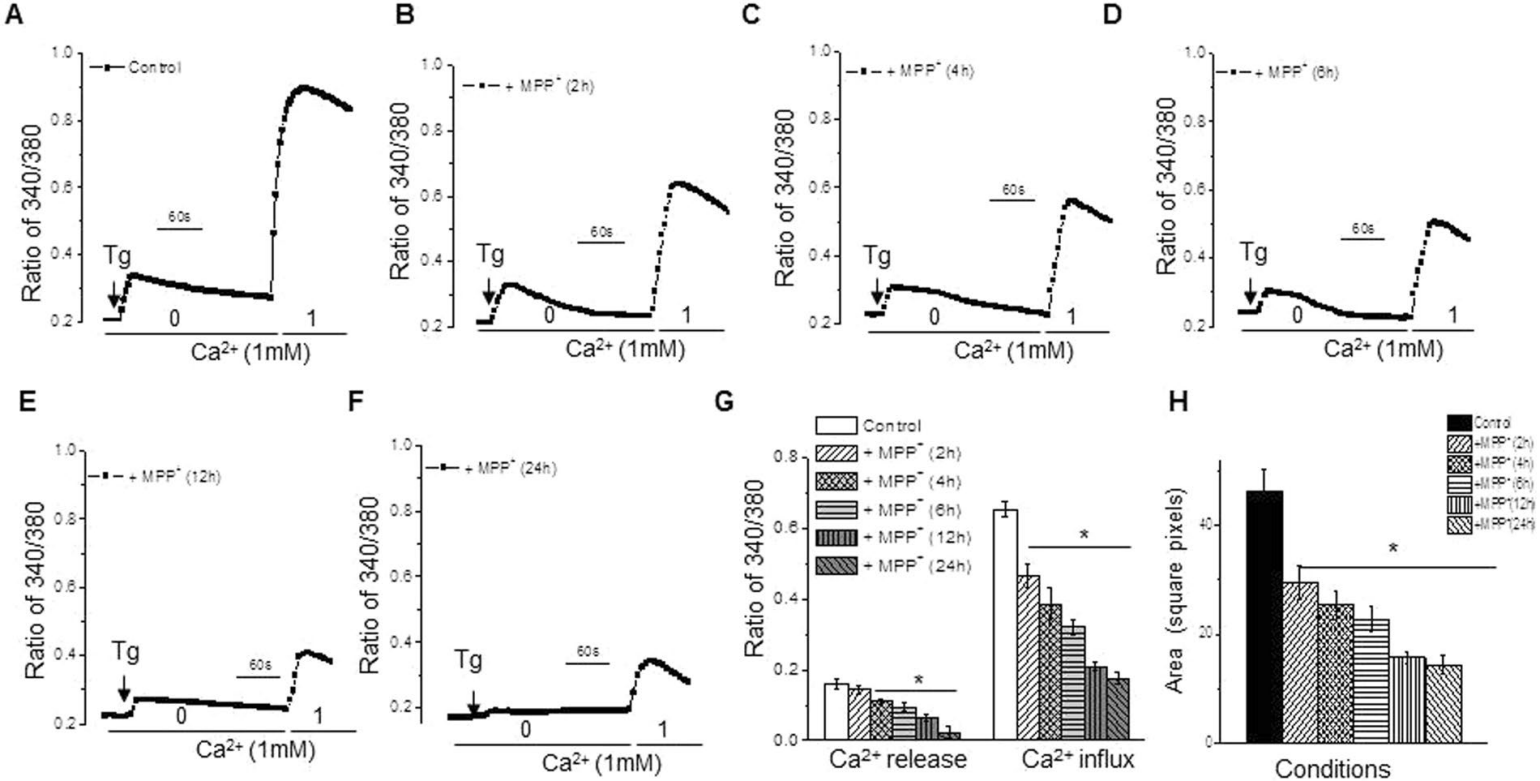
Prolonged treatment with MPP^+^ inhibited calcium release and influx in MSC derived dopaminergic neurons. Differentiated MSCs were exposed to 0 (**A**) or 500 µM MPP^+^ for 2 (**B**), 4 (**C**), 6 (**D**), 12 (**E**) or 24 (**F**) hours. Analog plots of fluorescence ratios (340/380) measured in the presence of 2 µM Thapsigargin, without (first peak of analog plot) or with (second peak of analog plot) 1 mM Ca^2+^ were determined. A graphical representation of 340/380 ratios (mean ± SE for each group) is represented in (**G**) and the area under the trace are shown in (**H**). *p value < 0.05, as compared with differentiated MSCs that were not treated with MPP^+^ from 50−100 cells in each conditions.

We next evaluated if MPP^+^ exposure can alter store-operated Ca^2+^ entry (SOCE) mechanisms in MSC-derived DNs. Addition of 2 µM Thapsigargin (Tg) in the absence of extracellular Ca^2+^ resulted in a slight increase in intracellular Ca^2+^ ([Ca^2+^]_i_) (first peak in Fig. 2A), which demonstrates release of Ca^2+^ from the ER in MSC-derived dopaminergic neuronal cytosol. Next, addition of 1 mM Ca^2+^ externally, initiated Ca^2+^ entry into the cytosol via SOCE, and this was recorded as a second peak (Fig. 2A) in these MSC-derived dopaminergic neurons. Treatment with 500 µM MPP^+^ for 2 h did not significantly affect the release of Ca^2+^ from the ER into the cytosol (first peak, Fig. 2B, and graphical representation in Fig. 2G, and area under the curve in Fig. 2H), but resulted in a decrease in SOCE (second peak, Fig. 2B), as compared with untreated MSC-derived DNs (Fig. 2A, and graphical representation in Fig. 2G). An extended treatment with MPP^+^ for 4, 6, 12 or 24 h not only showed a decrease in SOCE (second peak, Fig. 2A,C–G) but also in ER derived Ca^2+^ entry into the cytosol (first peak, Fig. 2A,C–G). This MPP^+^ mediated decrease in Ca^2+^ entry was observed only in MSC derived DNs and not in undifferentiated MSCs (data not shown). Together these results suggest that extended treatment with MPP^+^ results in diminished SOCE, which in turn decreases ER derived cytosolic Ca^2+^ that would lead to ER stress followed by the loss of dopaminergic neurons).

**Figure 3:**
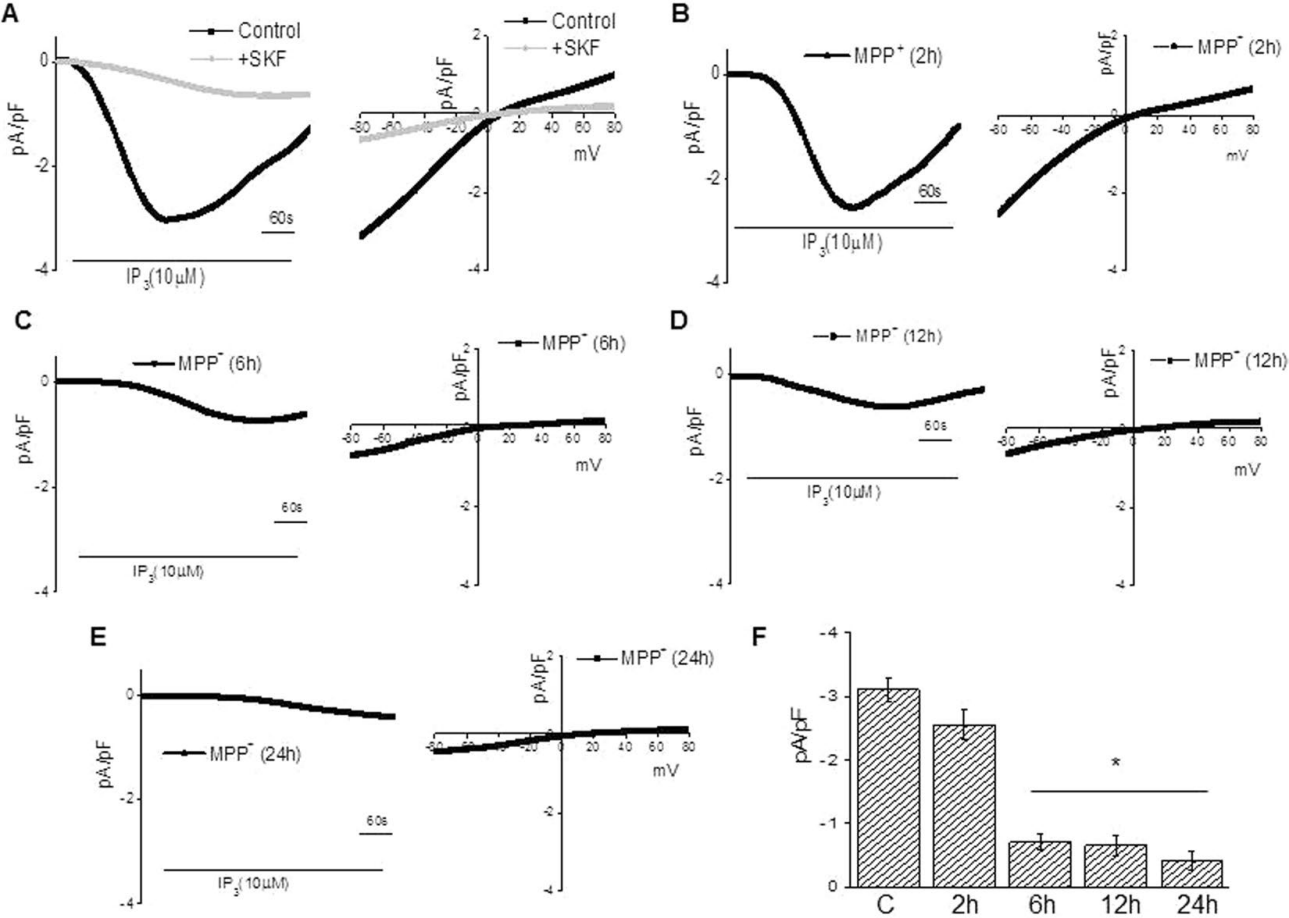
Prolonged treatment with MPP^+^ inhibited calcium currents in MSC-derived dopaminergic neurons. Ionositol 1, 4, 5-triphosphate (IP_3_) (10 µM) induced currents were measured in cells treated with 0 (**A**) or 500 µM MPP^+^ for 2 (**B**), 6 (**C**), 12 (**D**) or 24 (**E**) hours. Addition of SKF 96365 (50 µM) blocked the SOCE currents without changing the properties (**A**). The holding potential for current recordings was −80 mV. Relative current-voltage (I–V) curves (developed from maximum currents from 6–10 cells in each conditions) and expressed as mean ± SE for each group, are graphically represented in (**F**). *p value < 0.05, as compared with differentiated MSCs that were not treated with MPP^+^.

Next, we evaluated the properties of the SOCE currents in MSCs (both undifferentiated and differentiated MSCs). Importantly, the current properties were not changed upon differentiation (Fig. 3A); however addition of SKF to differentiated cells, blocked the SOCE currents in these cells. We next replaced Tg with a physiologically relevant second messenger inositol 1, 4, 5-triphosphate (IP_3_), for depleting the ER stores and initiating SOCE. Addition of IP_3_ in the patch pipet induced an inward current which was non-selective in nature, and reversed between 0 and −5 mV (Fig. 3A). The inward currents shown in Fig. 3 are recorded at a holding potential of −80 mV, and maximum peak currents were used for the tabulation of current intensity. The current-voltage (I–V) curves were made using a ramp protocol wherein current density was evaluated at various membrane potentials and plotted. The channel properties were similar to those previously observed with TRPC1 channels^45^, suggesting that TRPC1 could contribute to endogenous SOCE channel in these MSC-derived DNs. Importantly, treating MSC-derived DNs with MPP^+^ resulted in decreased Ca^2+^ entry (Fig. 3B–E), as compared with untreated MSC-derived DNs (Fig. 3A).While treatment with MPP^+^ for as less as 2 hours showed decreased Ca^2+^ entry, the reduction was significant with 6, 12 and 24 hour treatment (Fig. 3F graphically represents the results). However, the channel properties were not significantly different (as measured using I-V curves) under these conditions. Overall, the results presented in Figs 2 and 3 strongly suggest a significant decrease in MPP^+^ mediated SOCE, which could significantly alter Ca^2+^ homeostasis, thereby leading to the loss of DNs.

### MPP^+^ mediated decrease in SOCE may be attributed to reduced TRPC1 expression, and TRPC channel blockage leads to reduced dopamine release and neuronal cell viability

**Figure 4:**
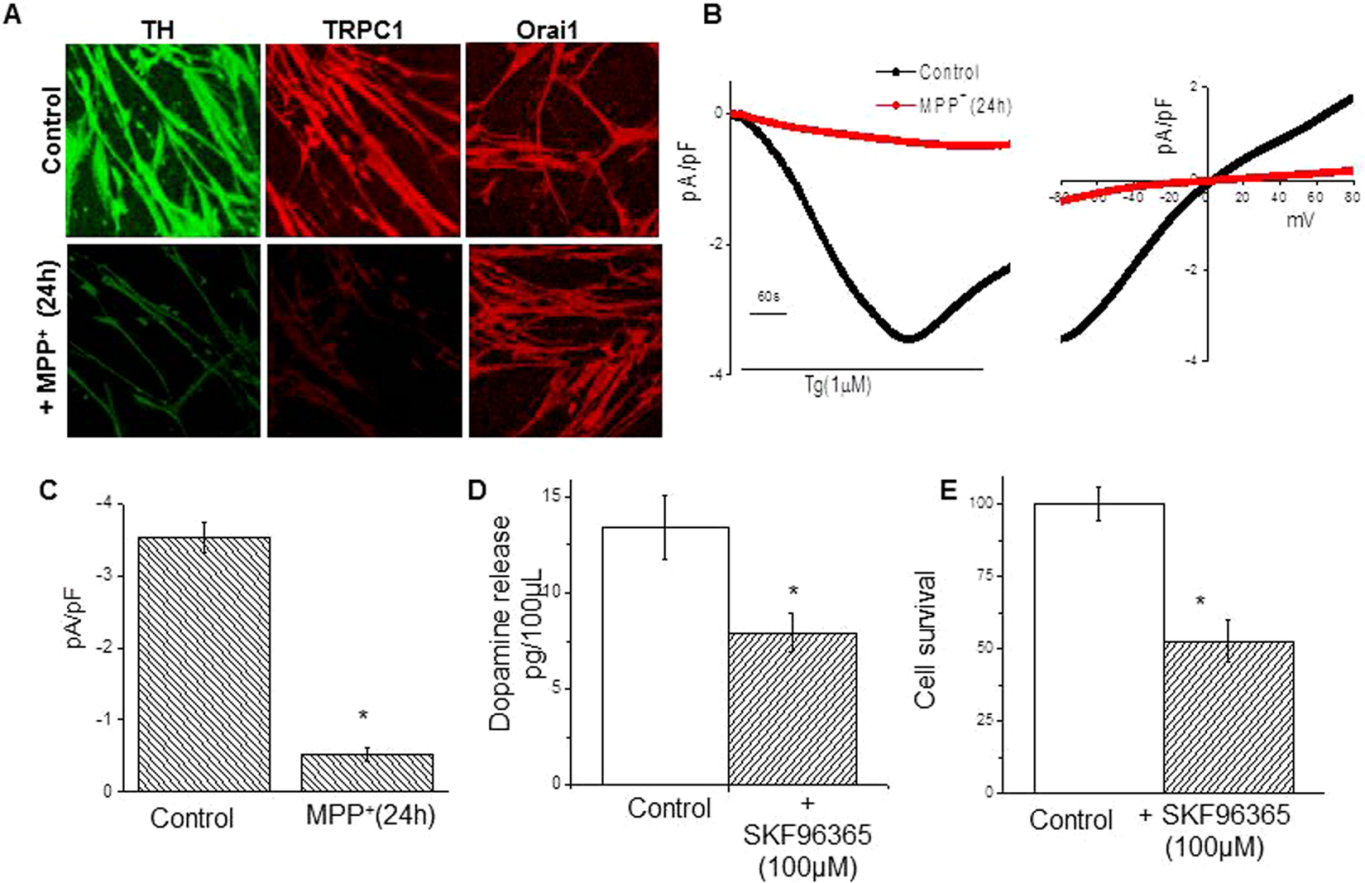
MPP^+^ inhibited TRPC1 expression, and TRPC reduction decreases SOCE, dopamine release and cell viability. Immunofluorescence was used to probe for endogenous TH (left) or TRPC1 (middle) or Orai1 (right) proteins in untreated (top) or MPP^+^ (500 µM for 24 hours; bottom) treated MSC-derived dopaminergic neurons (**A**). Tg (1 µM) induced average currents (from 7–9 cells) were measured in cells treated with 0 (**A**) or 500 µM MPP^+^ for 24 hours (**B**). The holding potential for current recordings was −80 mV. Relative current-voltage (I–V) curves (developed from maximum currents) and expressed as mean ± SE for each group, are graphically represented in C. ELISA was used to measure dopamine release from MSC-derived dopaminergic neurons that were either untreated or treated with SKF 96365 (100 µM for 30 mins), a non-specific TRPC channel blocker (**D**). MTT assay measured survival of MSC-derived dopaminergic neurons that were either untreated or treated with SKF 96365 (100 µM for 30 mins). *p value < 0.05, as compared with differentiated MSCs that were not treated with MPP^+^.

The data presented above strongly implicated that the endogenous calcium channel properties were similar to that of TRPC1. This prompted us to investigate the expression of TRPC1 protein in MPP^+^-treated (500 µM MPP^+^ for 24 hours) MSC-derived dopaminergic neurons (Fig. 4A, top panel). As ascertained via immunofluorescence, TRPC1 and Orai1 was localized in the neurites of the untreated MSC-derived dopaminergic neurons (Fig. 4A, top left). However, MPP^+^ treatment resulted in decreased TRPC1 staining, but no change in Orai1 staining, suggesting a reduced TRPC1 protein expression (Fig. 4A, middle and top right). Next, we compared the expression of TH protein in untreated MSC-derived dopaminergic neurons (Fig. 4A, lower left) and MPP^+^-treated MSC-derived dopaminergic neurons (Fig. 4A, lower right). Like TRPC1, TH was expressed in the neurites, and the immunostaining was reduced in MPP^+^-treated MSC-derived dopaminergic neurons, as compared with untreated cells. These results are consistent with our previous findings that TRPC1 protein is expressed in neuronal cells, and its expression is decreased upon MPP^+^ treatment^21^. Consistent with reduced expression of TRPC1, the Tg-induced inward Ca^2+^ currents were also decreased in MPP^+^ treated-MSC-derived dopaminergic neurons, as compared with untreated cells (Fig. 4B,C). Again, no change in the I-V curves was observed, and these currents were blocked by the addition of SKF 96365, a non-specific TRPC channel blocker, or gadolinium (Gd3^+^) (data not shown).

Next, we evaluated the role of TRPC mediated Ca^2+^ entry in dopamine release, and cell viability. For this purpose, we treated MSC-derived DNs with SKF 96365 to block the TRPC channel. As compared with untreated cells, SKF 96365 treated cells showed a significant decrease in dopamine release (Fig. 4D). Since we have previously shown that TRPC1 has a role in the protection of SH-SY5Y neuronal cells^21^, we questioned if loss of TRPC mediated Ca^2+^ entry would enhance neurodegeneration. MSC-derived dopaminergic neurons treated with SKF 96365 had significantly reduced survivability (~50% reduction) as compared with untreated cells (Fig. 4E). Similar reduction in dopamine release and cell viability was observed in MSC-derived dopaminergic neurons treated with MPP^+^ (data not shown). Taken together, these findings suggest that TRPC1 mediates SOCE in MSC-derived dopaminergic neurons and decreased TRPC1 function, either by the addition of neurotoxin (MPP^+^) or TRPC channel blocker (SKF 96365) leads to decrease in dopamine release and survival of DNs.

## Discussion

Dopaminergic Neurons (DNs) derived by differentiating stem cells under defined *in-vitro* conditions are an attractive therapeutic option for PD patients. In this study, we adopted a previously published approach to differentiate Mesenchymal Stem Cells (MSCs) into DNs using a 12-day protocol involving FGF-2, bFGF, SHH ligand and BDNF^43^. While MSC-derived DNs have been previously characterized for neuronal markers and electrophysiological properties^46^, we investigated the store-operated calcium entry (SOCE) mechanisms in these DNs under normal conditions, and upon exposure to environmental neurotoxin, 1-methyl, 4-phenyl pyridinium ion (MPP^+^). Our data implicated that like native neurons, MSC-derived DNs utilized SOCE mechanisms, which potentially involved TRPC1. Moreover, similar to native neurons, addition of neurotoxin (MPP^+^) decreased SOCE and TRPC1 protein expression in MSC-derived DNs. Blocking of TRPC channels in these MSC-derived DNs resulted in decreased dopamine release and cell viability. Overall, our report shows that MSC-derived DNs are functional with regard to SOCE mechanisms, and MPP^+^ exposure dysregulates calcium signaling in these neurons, making them vulnerable to neurodegeneration.

Several cellular sources have been investigated for the potential to act as renewable supply of transplantable DNs for PD, including fetal brain tissue^47,48^, embryonic stem cells^49,50^, neural precursor stem cells^51–53^, induced pluripotent stem cells^54^ and MSCs^28–41^. The use of MSCs for cell-based therapy is viewed upon as an attractive approach, since these cells can be (a) easily isolated from small aspirates of bone marrow, (b) easily expanded in culture with low tumorigenicity and teratoma formation, (c) display immunosuppressive properties that are advantageous for transplantation^55,56^, and (d) may allow autolgous transplantation under ideal settings^27^. Additionally, several studies demonstrated expression of dopaminergic neuronal markers by un-differentiated hMSCs^40,57–59^, thereby suggesting their neural pre-disposition, and provide support for their differentiation capacity to functional DNs, and for their use in cell-based therapy for PD. Lastly, MSCs injected into the brains of PD patients are likely to function not only by differentiating into DNs, but also by secreting cytokines and growth factors that may function in a paracrine manner to promote endogenous neuronal growth, decrease apoptosis, reduce levels of free radicals, encourage synaptic connection from damaged neurons and regulate inflammation^60,61^.

Dopaminergic neuronal differentiation of rodent and human MSCs in an *in-vitro* setting has been attempted by several researchers^28–41,62–64^. The majority of these studies employed the addition of extrinsic differentiation inducing factors, such as cytokines, small molecules, gene transfection, conditioned medium, and co-culturing with cells such as astrocytes^27^. These studies have exhibited varying degrees of success involving generation of TH-positive neurons, physiological properties, dopamine secretion, and symptomatic improvement in rodent models of PD. Several earlier differentiation studies were limited in their potential for clinical translation, as they utilized gene transfection^29^, conditioned medium or co-culture systems with other cell types such as astrocytes^28^. Earlier differentiation protocols utilizing a combination of cytokines or small molecules weren’t efficient in terms of the % of cells expressing dopaminergic neuronal markers or lacked demonstration of electrophysiological properties which characterized neurons. However, the approach that we utilized for our study has been shown to result in ~70% cells expressing tyrosine hydroxylase (enzyme required for dopamine synthesis), and >90% of cells expressed neuronal markers, NeuN and beta-III-tubulin^46^. Additionally, these cells showed the presence of voltage-gated Ca^2+^ channels, exhibited inward spontaneous post-synaptic currents, and secreted dopamine in response to Ca^2+^ depolarization^46^. These findings indicated the presence of functional neuronal circuits in these neurons, similar to native neurons.

Mitochondrial dysfunction in DNs has been implicated as an important factor in the pathogenesis of sporadic and familial PD, and leads to apoptotic death of these neurons. This represents a potentially valuable therapeutic target in these patients^65^. Mitochondria are important cellular organelles as ATP producers and Ca^2+^ signal regulators. The environmental neurotoxin, MPTP, functions as an inhibitor of mitochondrial respiratory chain Complex 1, causing Parkinson-like symptoms. MPTP is converted to MPP^+^ by glial cells and taken up by DNs selectively through dopamine transporter (DAT), which is exclusively expressed in DNs^12^. Our data shows that unlike un-differentiated hMSCs, the differentiated hMSCs (DNs) express DAT. The fact that MSC-derived DNs are sensitive to the actions of MPP^+^, as demonstrated by reduced SOCE, TRPC1 and TH expression suggests that MSC-derived DNs not only express DAT, but are also capable of taking up MPP^+^. Thus, we propose that this MSC-derived dopaminergic neuronal differentiation model can be used to study mitochondrial homeostasis in the presence of MPP^+^.

MPP^+^ was found to dysregulate Ca^2+^ homeostasis in our previous studies utilizing neuroblastoma cell line (SH-SY5Y cells)^21^. We demonstrated that endogenous SOCE, which is critical for maintaining ER Ca^2+^ levels (as prolonged neurotoxin treatment showed a decrease in ER calcium levels as well), is dependent on TRPC1 activity. While the molecular identity of SOCE channels remains ambiguous, our study provided evidence that TRPC1, even if not direct component of SOCE channel, did regulate SOCE^21^ in these cells. TRPC1 mediated Ca^2+^ entry was found to be essential for maintaining ER Ca^2+^ stores, and consequent prevention of ER stress and unfolded protein response (UPR) is protective against neurodegeneration. Most importantly, this study demonstrated that in SH-SY5Y neuroblastoma cells, MPP^+^ treatment decreased TRPC1 expression, TRPC1 interaction with the SOCE modulator stromal interaction molecule 1 (STIM1), and Ca^2+^ entry into the cells. These results were instrumental in showing the mechanism involving SOCE and TRPC1 channel, by which a widely used model compound (MPP^+^) causes neurodegeneration by altering Ca^2+^ homeostasis and inducing ER stress in PD^21^. While the mechanism of MPP^+^ induced neurodegeneration was exciting, the study was limited in the fact that it utilized a neuroblastoma cell line originally derived from a malignant tumor^66^, which is far from mimicking native neurons.

In the current report, we tested the MPP^+^ induced Ca^2+^ dyshomeostasis in the physiologically relevant model MSC-derived DNs. Like in SH-SY5Y cells, MPP^+^ treatment decreased TRPC1 expression and Ca^2+^ entry into the cells in a time-dependent manner. The endogenous SOC showed I-V relationships that were similar to those observed for TRPC1-dependent currents^45^. TRPC blockage with an inhibitor compound decreased dopamine release and MSC-derived DN cell viability. These results reinforce our previous findings that Ca^2+^ homeostasis and ER Ca^2+^ levels, maintained with the help of SOCE, and potentially involving TRPC1, are neuroprotective. Exposure to MPP^+^ may cause neurodegeneration by altering SOCE and TRPC1 mediated Ca^2+^ homeostasis, which may induce ER stress, UPR and ultimately lead to neuronal death. These results underscore the fact that MSC-derived DNs characterized in the current report are similar to native neurons in expressing TRPC1 channels, SOCE mechanisms, and are sensitive to PD model compound MPP^+^.

In summary, our study provides the first comprehensive analysis of SOCE mechanisms in MSC-derived DNs, and the effect of an environmental neurotoxin, MPP^+^, on SOCE mechanisms in these neurons. We have shown that these neurons express neuronal markers, electrophysiological properties, SOCE mechanisms, TRPC1 protein expression and are sensitive to MPP^+^, like native neurons. This broadens the prospect of utilizing this differentiation protocol for regenerative therapy in PD patients. By investigating the effect of MPP^+^ on MSC-derived DNs, we propose a mechanism by which MPP^+^ is possibly neurodegenerative, by dysregulating SOCE mediated Ca^2+^ homeostasis, which potentially involves TRPC1. This serves as an example of how MSC-differentiation to DN described here, may serve as a model for neuronal development and disease modeling for PD. Besides generating DNs similar to native neurons, the length of this model (12 days) is apt for analyzing genetic and epigenetic changes associated with dopaminergic neuronal differentiation, under physiological and PD relevant pathological conditions.

## Electronic supplementary material

Dataset 1
