# Supplementary material for: MPP+ decreases store-operated calcium entry and TRPC1 expression in Mesenchymal Stem Cell derived dopaminergic neurons
Source: Sci Rep. 2018 Aug 6;8:11715. doi: 10.1038/s41598-018-29528-x (PMC6079049; doi:10.1038/s41598-018-29528-x)
Supplement: Supplementary file 1 — Dataset 1 [file 41598_2018_29528_MOESM1_ESM.pdf]

MPP$^+$ decreases store-operated calcium entry and TRPC1 expression in Mesenchymal Stem Cell derived dopaminergic neurons

Yuyang Sun[3], Senthil Selvaraj[1], Sumali Pandey[2], Kristen M. Humphrey[3], James D. Foster[1], Min Wu[1], John A. Watt[1], Brij B. Singh[4] and Joyce E. Ohm[3]

[1]Department of Biomedical Sciences, School of Medicine and Health Sciences, University of North Dakota, Grand Forks, North Dakota 58203, USA

[2]Biosciences Department, Minnesota State University, Moorhead, Moorhead, MN

[3]Department of Cancer Genetics and Genomics, Roswell Park Cancer Institute, Buffalo, NY 14263

[4]School of Dentistry, UT Health Science Center San Antonio, TX 78229

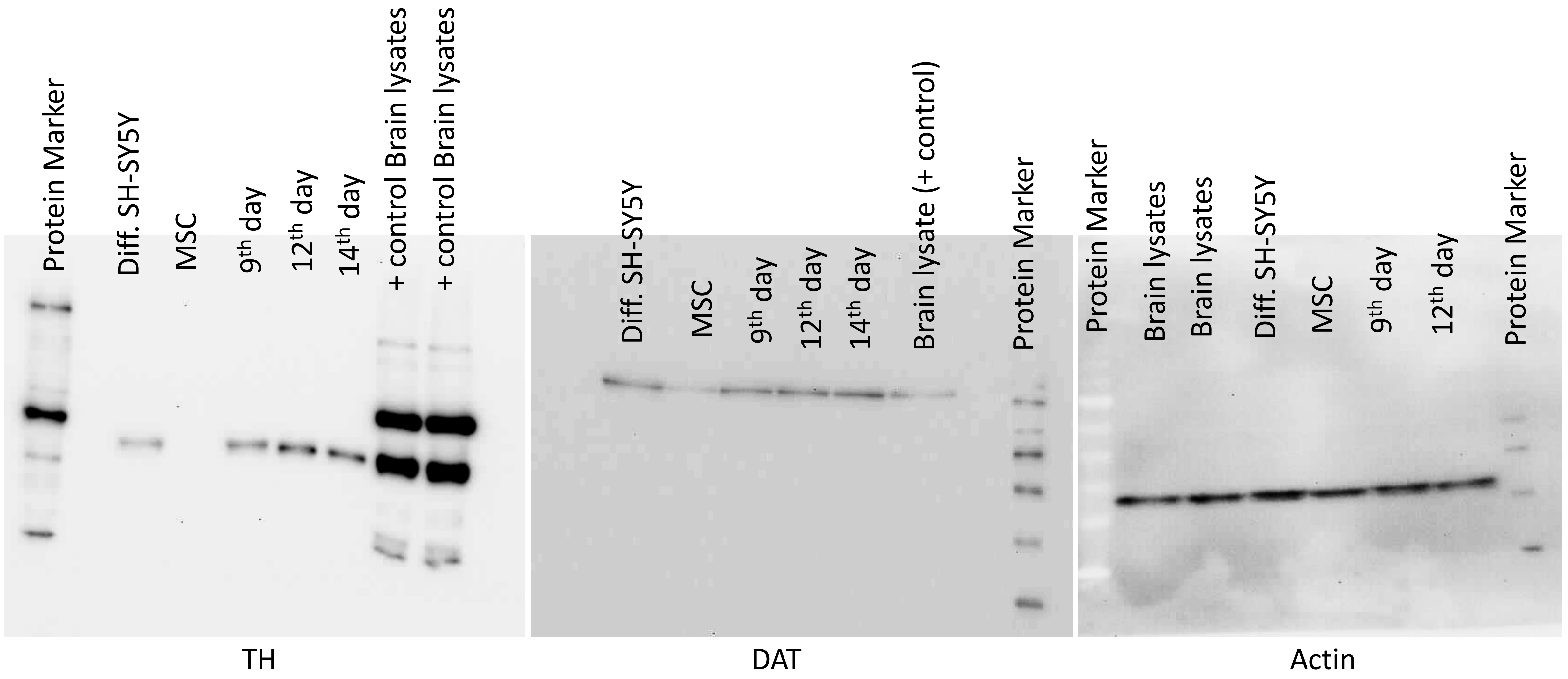
Protein Marker
Diff. SH-SY5Y
MSC
9th day
12th day
14th day
+ control Brain lysates
+ control Brain lysates
TH
Diff. SH-SY5Y
MSC
9th day
12th day
14th day
Brain lysate (+ control)
Protein Marker
DAT
Protein Marker
Brain lysates
Brain lysates
Diff. SH-SY5Y
MSC
9th day
12th day
Protein Marker
Actin
